# Whole-Genome Sequencing and Virulome Analysis of Escherichia coli Isolated from New Zealand Environments of Contrasting Observed Land Use

**DOI:** 10.1128/aem.00277-22

**Published:** 2022-04-20

**Authors:** Adrian L. Cookson, Jonathan C. Marshall, Patrick J. Biggs, Lynn E. Rogers, Rose M. Collis, Megan Devane, Rebecca Stott, David A. Wilkinson, Janine Kamke, Gale Brightwell

**Affiliations:** a Food Systems Integrity, AgResearch Limited, Hopkirk Research Institute, Massey University, Palmerston North, New Zealand; b mEpiLab, School of Veterinary Science, Massey University, Palmerston North, New Zealand; c School of Mathematics and Computational Sciences, Massey University, Palmerston North, New Zealand; d School of Natural Sciences, Massey University, Palmerston North, New Zealand; e New Zealand Food Safety Science and Research Centre, Massey University, Palmerston North, New Zealand; f Environmental Science and Research Limited, Christchurch, New Zealand; g National Institute of Water and Atmospheric Research, Hamilton, New Zealand; h Horizons Regional Council, Palmerston North, New Zealand; Centers for Disease Control and Prevention

**Keywords:** *Escherichia*, STEC, virulome, whole-genome sequencing, environment, phylogroup, environmental microbiology

## Abstract

Generic Escherichia coli is commonly used as an indicator of fecal contamination to assess water quality and human health risk. Where measured E. coli exceedances occur, the presence of other pathogenic microorganisms, such as Shiga toxin-producing E. coli (STEC), is assumed, but confirmatory data are lacking. Putative E. coli isolates (*n* = 709) were isolated from water, sediment, soil, periphyton, and feces samples (*n* = 189) from five sites representing native forest and agricultural environments. Ten E. coli isolates (1.41%) were *stx*_2_ positive, 19 (2.7%) were *eae* positive, and *stx*_1_-positive isolates were absent. At the sample level, *stx*_2_-positive E. coli (5 of 189, 2.6%) and *eae*-positive isolates (16 of 189, 8.5%) were rare. Using real-time PCR, these STEC-associated virulence factors were determined to be more prevalent in sample enrichments (*stx*_1_, 23.9%; *stx*_2_, 31.4%; *eae*, 53.7%) and positively correlated with generic E. coli isolate numbers (*P* < 0.05) determined using culture-based methods. Whole-genome sequencing (WGS) was undertaken on a subset of 238 isolates with assemblies representing seven E. coli phylogroups (A, B1, B2, C, D, E, and F), 22 Escherichia marmotae isolates, and 1 Escherichia ruysiae isolate. Virulence factors, including those from extraintestinal pathogenic E. coli, were extremely diverse in isolates from the different locations and were more common in phylogroup B2. Analysis of the virulome from WGS data permitted the identification of gene repertoires that may be involved in environmental fitness and broadly align with phylogroup. Although recovery of STEC isolates was low, our molecular data indicate that they are likely to be widely present in environmental samples containing diverse E. coli phylogroups.

**IMPORTANCE** This study takes a systematic sampling approach to assess the public health risk of Escherichia coli recovered from freshwater sites within forest and farmland. The New Zealand landscape is dominated by livestock farming, and previous work has demonstrated that “recreational exposure to water” is a risk factor for human infection by Shiga toxin-producing Escherichia coli (STEC). Though STEC isolates were rarely isolated from water samples, STEC-associated virulence factors were identified more commonly from water sample culture enrichments and were associated with increased generic E. coli concentrations. Whole-genome sequencing data from both E. coli and newly described *Escherichia* spp. demonstrated the presence of virulence factors from E. coli pathotypes, including extraintestinal pathogenic E. coli. This has significance for understanding and interpreting the potential health risk from E. coli where water quality is poor and suggests a role of virulence factors in survival and persistence of E. coli and *Escherichia* spp.

## INTRODUCTION

Escherichia coli is a common inhabitant of the gastrointestinal microbiota of mammals and birds (1) and is used routinely as a predictor of fecal contamination for water quality assessment purposes (2, 3) and as a proxy for other excreted waterborne pathogens less amenable to culture and detection (4). Sites with contrasting levels of observed land use are often sampled to provide an indication of E. coli levels (3, 5) and to support the development of risk-based guidelines to protect human health. Sites affected by urban point-source discharge or agricultural surface runoff frequently have increased levels of E. coli in water samples that are of fecal origin (6). On the other hand, New Zealand native habitats, actively managed to enhance native biodiversity and protect endemic species through intensive pest management strategies, offer a unique opportunity to study the prevalence of pathogenic E. coli and undertake analysis of environmental E. coli in relatively untouched pristine landscapes. These environments offer suitable sites from which baseline levels of E. coli can be measured and to provide information on the distribution, prevalence, and virulence factors of E. coli using high-resolution methods such as whole-genome sequencing (WGS) of isolates.

Most generic E. coli isolates found in freshwater and the surrounding environment are nonpathogenic. However, some pathogenic types of E. coli, such as Shiga toxin-producing E. coli (STEC), defined as E. coli that possesses *stx*_1_ and/or *stx*_2_ allelic variants, are associated with significant outbreaks of disease and may cause serious illness in young children, the elderly, and the immunocompromised (7). A previous case-control study in New Zealand identified “contact with recreational waters” as a significant environmental risk factor associated with human diarrheal disease caused by STEC (8), but little work has been undertaken to confirm and identify *stx*-positive isolates from such samples, and the potential health risk from water contact has not been explored fully. Several overseas STEC-outbreak investigations have identified the same STEC isolates from recreational lake water studies and those recovered from STEC-infected clinical cases (9, 10), but the public health risk assessments of the hazard that STEC poses in freshwater rivers and streams is less well understood (11–13) due to the limitations of culture-based approaches. Therefore, the aim of this work was to (i) examine the prevalence of STEC and (ii) undertake WGS analysis of generic E. coli isolated from environmental samples (deposited animal and avian feces, water, soil, sediment, and periphyton, biofilm material attached to submerged surfaces) sourced from sites of contrasting observed land use (native forest, dairy, or sheep and beef farming) to examine the spatiotemporal distribution and identify the genomic frequency of virulence traits (virulome) associated with human disease.

## RESULTS

### Site visits, sampling, and bacterial isolation.

Over the 11-month period, 28 site visits occurred; sites 1, 2, and 5 were each visited on six occasions, and sites 3 and 4 were visited on five occasions (Table S1). Unlike site 1, where activities are undertaken to enhance native biodiversity through the trapping and elimination of introduced mammal pests, the other four sites are associated with low levels of endemic biodiversity due to pastoral farming activities. Opportunistic fecal samples and soil and water samples were obtained from each site, along with sediment and periphyton, when water levels permitted. Sediment was not collected from site 2, as the river substrata consisted of large rocks and pebbles from which periphyton samples were obtained. Additional duplicate water samples collected on the same day were provided by Horizons Regional Council staff during routine monthly monitoring for additional water quality parameters from site 2 (four occasions) and site 4 (four occasions). For site 5, multiple water (*n* = 3), sediment (*n* = 3), soil (*n* = 2), and opportunistic fecal samples were collected on each of the six sampling visits to examine the persistence and survival of E. coli through the length of the wetland for a separate study not reported here. Generally, four separate presumptive E. coli isolates were recovered for subsequent analysis from each sample.

### Subtyping and *Escherichia* isolate phylogeny.

**FIG 1:**
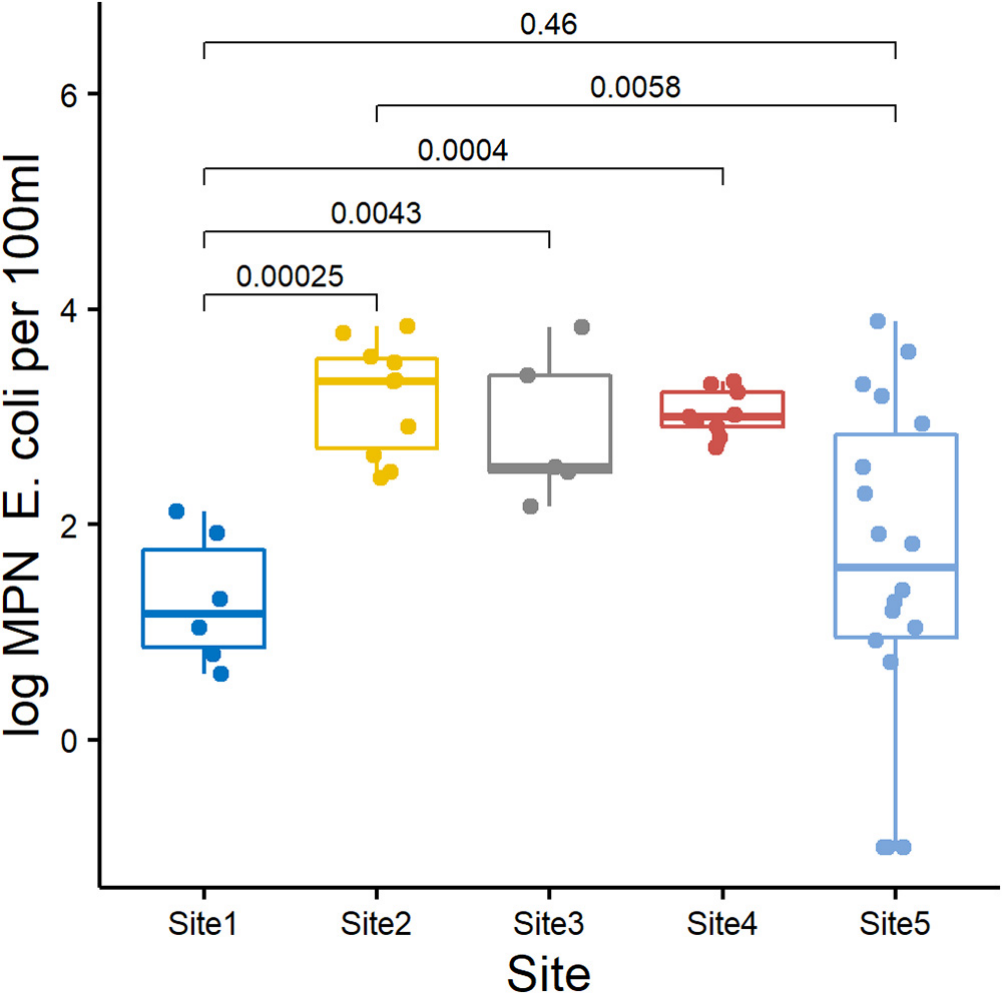
Box and whisker plot of log_10_-transformed E. coli (MPN/100 mL) water counts from each site (site 1: 6 visits, 6 water samples; site 2: 6 visits, 10 water samples; site 3: 5 visits, 5 water samples; site 4: 5 visits, 9 water samples; site 5: 6 visits, 17 water samples). Samples with 0 MPN/100 mL were converted to 0.1 (log = −1). The boxes show median values and span lower to upper quartiles, the whiskers show the highest and lowest values within 1.5 times the interquartile range, and dots beyond the whiskers show potential outliers. The Wilcoxon rank sum test was used to compare counts from site 1 paired with the other four sites, and site 2 with site 5 (*P* value). All other pairwise comparisons were not significant (*P* > 0.05).

E. coli isolates were isolated from all sites (Table 1) and most samples (173 of 189, 91.5.0%), including all periphyton (*n* = 26) and most feces (45 of 47, 95.7%), sediment (33 of 35, 94.3%), water (44 of 47, 93.6%), and soil (24 of 34, 70.6%) samples. E. coli counts from water averaged 1,236 most probable number (MPN) per 100 mL, and numbers in water samples from bush headwater stream site 1 and wetland site 5 were lower than those in higher order waterways (Fig. 1). Compared to those isolated from soil samples (average 29.8 MPN per gram dry weight), E. coli isolates were commonly isolated in greater numbers from aquatic sediment samples (average 89.1 MPN per gram dry weight, *P* = 0.019, analysis of variance [ANOVA]).

**TABLE 1:** New Zealand sample site details where water, soil, sediment, periphyton, and fecal samples were obtained for this study

Sample site	Value for site:				
	Pūkaha mount bruce	Makakahi river, hamua bridge	Mākirikiri stream	Mangaterā stream	Toenepi constructed wetland
GPS	40° 44′ 2.774′′ S 175° 38′ 15.802′′ E	40° 33′ 55.458′′ S 175° 44′ 45.909′′ E	40° 13′ 40.48′′ S 176° 05′ 46.30′′ E	40° 13′ 41.11′′ S 176° 05′ 49.42′′ E	37° 43′ 4.8′′ S 175° 35′ 7.511′′ E
Land use	Native forest	Sheep and beef farming	Small native forest stand and dairy, sheep, and beef farming	Dairy, sheep, and beef farming	Dairy farming (3 cows/ha)
Elevation (m)	354	171	159	159	49
Avg rainfall (mm)	1,500	1,492 (Eketahuna)	1,054 (Dannevirke)	1,054 (Dannevirke)	1,235 (Tauwhare)
Catchment population	None	Eketahuna township popn 441 (distance 9.5 km)	<100	Dannevirke township popn 5,200 (distance 2 km)	<10
Stream order	Second	Fourth	Third	Fourth	Tile drainage
Width (m)	Min 1.31, max 2	16 (estimate)	Min 1.17, max 3.3	10 (estimate)	2 surface-flow cells in series ~5 m wide by 26 m long
Depth (cm)	Min 7, max 17	NR^*a*^	Min 5.8, max 33.5	Min 14.25, max 23^*b*^	Inflow: min 9.7, max 27.0; bund: min 12, max 26.0; outlet: min 7.0, max 22.0
E. coli counts					
Water^*c*^	Avg 43 (min 4; max 133)	Avg 3,887 (min 272, max 8,130)	Avg 2,016 (min 145, max 6,867)	Avg 916 (min 213, max 1,989)	Avg 940 (min 0, max 7,710)
Sediment^*d*^	Avg 5 (min 0; max 30)	NR	Avg 169 (min 60, max 484)	Avg 393 (min 39, max 1,564)	Avg 13 (min 0, max 106)
Soil^*d*^	Avg 1 (min 0; max 7)	Avg 27 (min 0, max 71)	Avg 63 (min 0, max 315)	Avg 80 (min 0, max 387)	Avg 15 (min 0, max 129)

a NR, not recorded, site too deep to measure safely.

b Depth could not be measured on two visits due to colored water and streambed not visible.

c MPN per 100 mL water.

d MPN per gram dry weight.

Over the 28 separate sampling visits, 709 presumptive E. coli isolates were isolated from the five sites. Real-time PCR (RT-PCR) was undertaken to identify the presence of STEC-associated genes encoding Shiga toxins and the protein intimin, involved in the formation of attaching and effacing lesions on epithelial cells, using primer/probe combinations specific for *stx*_1_, *stx*_2_, and *eae*, respectively. Ten E. coli isolates recovered from 5 samples (bovine feces, two water, soil, and periphyton) were *stx*_2_ positive, and 18 E. coli isolates from 16 samples (six water, five feces, three sediment, and two periphyton) were *eae* positive. Although *stx*_2_-positive E. coli isolates were isolated from only 2.6% (5 of 189) of samples using culture-dependent methods, 29.1% (55 of 189) of enriched environmental samples were *stx*_2_ positive using RT-PCR. Similarly, *eae*-positive E. coli isolates were recovered from 16 samples, but 54.0% (102 of 189) of enrichment samples were positive using RT-PCR. No *stx*_1_-positive E. coli isolates were isolated, but 23.8% (45 of 189) of enriched samples were *stx*_1_-positive using RT-PCR. No E. coli isolates were identified that were both *stx* and *eae* positive. Generally, linear mixed effects models using RT-PCR data to determine the presence or absence of virulence factors revealed that the *stx*_1_, *stx*_2,_ and *eae* virulence factors were more prevalent in environmental sample enrichments obtained from sites 2, 3, and 4, associated with large freshwater catchments where agriculture was the primary observed land use, than in enrichments from sites 1 (land-use native bush) and 5 (dairy farm wetland with small 2.6 ha catchment) (Table 2). Likewise, using the RT-PCR data describing the presence or absence of *stx*_1_, *stx*_2,_ and *eae* virulence factors, linear mixed effects models showed that an increased prevalence of *stx*_1_ (*P* = 0.001), *stx*_2_ (*P* = 0.0001), and *eae* (*P* = 0.0048) alleles from water (enrichment culture of 100 mL of filtered water) was associated with increased generic E. coli MPN/100 mL counts.

**TABLE 2:** A binomial mixed effects model was applied to real-time PCR data incorporating presence (*C*_q_ <35) or absence (*C*_q_ >35) of the *stx*_1_, *stx*_2_, and *eae* target virulence alleles from individual environmental sample enrichments^*a*^

Outcome variable	Fixed effect (site no.)	OR^*b*^	95% CI	P-value^*c*^
*stx*_1_	1	0.02	0.002–0.19	0.0006***
	2	82.41	6.66–1019.99	0.0006***
	3	22.19	1.86–265.23	0.014*
	4	27.27	2.37–314.17	0.008**
	5	3.82	0.33–43.81	0.281
*stx*_2_	1	0.12	0.44–0.35	<0.0001***
	2	13.3	3.53–50.31	0.0001***
	3	5.5	1.51–20.02	0.01**
	4	5.6	1.61–19.42	0.007**
	5	2.31	0.70–7.57	0.168
*eae*	1	0.33	0.15–0.72	0.005**
	2	15.3	1.97–59.01	<0.0001***
	3	8.78	2.67–28.89	0.0003***
	4	8.56	2.79–26.28	0.0002***
	5	2.00	0.78–5.16	0.147

a Sample site was the fixed effect variable and visit was included as a random effect. *, *P* < 0.05; **, *P* < 0.01; ***, *P* < 0.001. CI, confidence interval.

b OR, Odds ratio.

c P-value, corresponds to the null hypothesis OR = 1 (i.e. no effect) in the population.

RT-PCR targeting the *uid*A gene was able to provisionally identify 674 of 709 (95.1%) isolates as E. coli. The remaining 35 isolates (4.9%) from which no *uid*A amplicon was generated were identified as cryptic *Escherichia* clades (1 clade IV, E. ruysiae, and 34 clade V, E. marmotae) using clade-specific PCR (14) and were isolated from 21 samples (water, *n* = 8; soil, *n* = 2; sediment, *n* = 5; periphyton, *n* = 5; and avian feces, *n* = 1) across all five sites. Importantly, these newly described *Escherichia* spp. were less prevalent in fecal samples than E. coli (*P* = 0.007, Fisher’s exact test).

Bacterial subtyping targeting a 284-bp fragment of the hypervariable *gnd* gene separated the 709 isolates into 212 distinct *gnd* sequence types (gSTs). A total of 238 isolates were selected for WGS (Table S2) and were represented by 79 distinct gSTs, including 215 E. coli, a single *E. ruysiae*, and 22 *E. marmotae* isolates (Table S3). Isolates for WGS were selected from all five sites comprising between 23.9% of the total isolates collected from site 5 (58 of 243 isolates) and 44.4% from site 3 (48 of 108 isolates). The proportion of isolates selected from each of the five sites for WGS analysis (*n* = 238) ranged from 15.1% (36 of 238 isolates from site 2) to 24.4% (58 of 238 isolates from site 5). Isolates were chosen for WGS analysis according to (i) identification of gSTs common to different samples, sites, or site visits, (ii) those from animal fecal samples for fecal source tracking purposes, (iii) non-E. coli, and (iv) isolates from which STEC-associated virulence factors *stx*_2_ and *eae* were identified using RT-PCR.

WGS data from the 238 isolates were processed using the Nullarbor (v2.0) pipeline and the Center for Genomic Epidemiology output to evaluate the core genome, virulence genes, and antibiotic resistance genes. *De novo* assembled genomes varied in size from 4.48 Mb to 5.38 Mb (average 4.94 Mb, SD 0.179 Mb) (Table S2) and were separated, using the ClermonTyper web interface, into at least seven E. coli phylogroups, 25 A (10.5%), 126 B1 (52.9%), 30 B2 (12.6%), 2 C (0.8%), 22 D (9.25%), 9 E (3.8%), 1 F (0.45%), together with the 1 *E. ruysiae* (clade IV, 0.45%) and 22 *E. marmotae* (clade V, 9.25%) isolates (Table 3, Table S3). Individual phylogroups (A, B1, B2, D, and E) were recovered from most sites and individual sample types, except phylogroup B2, which was notably absent in the WGS panel from site 5 (Table S3).

**TABLE 3:** Summary of E. coli phylogroups and non-E. coli *Escherichia* species isolates from different environmental sample types

Sample type	Total no. of isolates	No. of isolates by phylogenetic group^*a*^							
		A	B1	B2	C	D	E	F	Non-E. coli^*b*^
Feces	66	6	34	14	1	8	1	0	2
Avian	23	2	8	7	0	4	0	0	2
Nonavian	43	4	26	7	1	4	1	0	0
Periphyton	35	3	16	6	1	3	1	0	5
Sediment	37	5	20	2	0	4	1	0	5
Soil	23	0	18	1	0	1	1	0	2
Water	75	11	38	7	0	6	4	1	9
									
Overall total	238	25	126	30	2	22	9	1	23

a E. coli phylogroup was determined using the ClermonTyper web interface (80).

b Non-E. coli species identified were *E. marmotae* (cryptic clade V, *n* = 22) and *E. ruysiae* (cryptic clade IV, *n* = 1).

**FIG 2:**
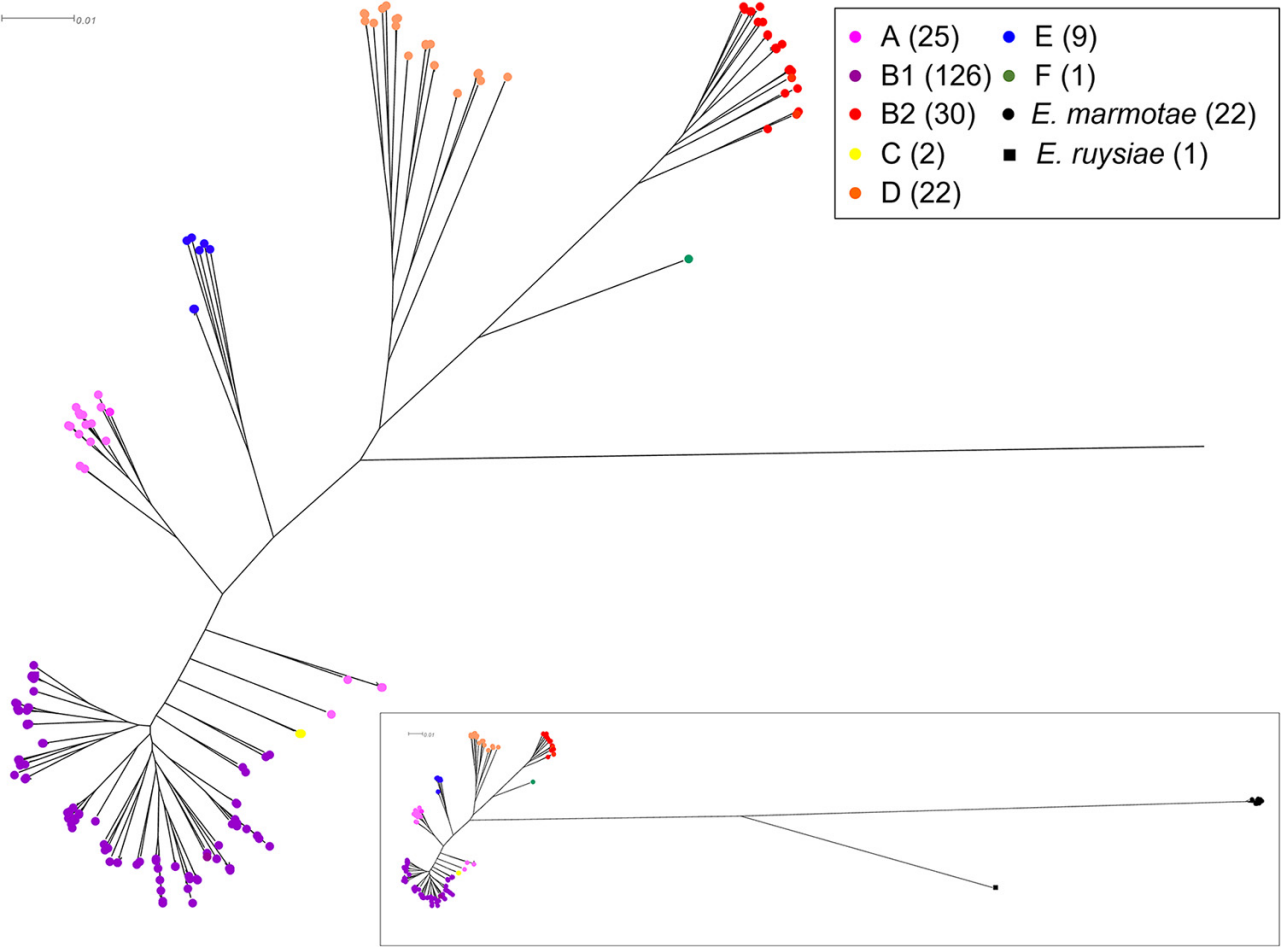
Maximum-likelihood tree visualized in SplitsTree (v.4.14.8) (76) of core genome single nucleotide polymorphism phylogenetic analysis of *Escherichia* species isolates (*n* = 238) from water, soil, sediment, periphyton, and feces. E. coli isolates were examined using Snippy (v.4.2.1) (74) with E. coli IAI39 (accession CU928164) as the reference genome (removed from tree). Clustering broadly separated E. coli isolates into phylogroups A, B1, C, and E, which are more commonly associated with intestinal infections, and B2, D, and F, more frequently noted as extraintestinal pathogenic E. coli. The newly described species *E. ruysiae* and *E. marmotae* were clustered separately from E. coli, forming their own monophyletic group (inset).

Single nucleotide polymorphism (SNP) analysis using 402,048 SNPs (8.1% of average genome size, 4.94 Mb) further confirmed the separation of isolates according to phylogroup and *Escherichia* species (Fig. 2). Clustering using maximum-likelihood phylogenetic trees broadly separated E. coli into phylogroups A, B1, C, and E, which are more commonly associated with intestinal colonization, while B2, D, and F clustered closely and are noted more frequently as extraintestinal pathogenic E. coli (15). The two phylogroup C isolates were positioned between phylogroups A and B1, and the single phylogroup F isolate was emergent from the B2 branch. The newly described species *E. ruysiae* and *E. marmotae* clustered separately from E. coli, forming their own monophyletic group.

**FIG 3:**
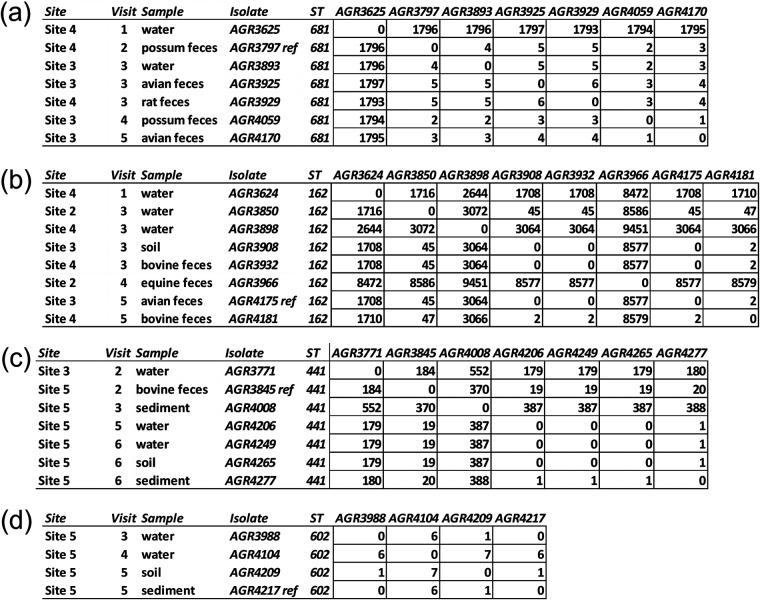
Distance matrix of E. coli with identical *gnd* sequence types was determined using core genome single nucleotide polymorphism phylogenetic analysis (Snippy v.4.2.1) (74) of isolates and internal reference sequence to generate pairwise SNP matrices. (a) gST535, (b) gST161, (c) gST251, and (d) gST395.

Using pubMLST (16) and the Achtman seven-locus MLST scheme, we identified 79 different sequence types (ST) from WGS data; ST-10 (*n* = 12), ST-154 (*n* = 12), and ST-162 (*n* = 17) were the most abundant, representing 9, 4, and 8 gSTs, respectively (Table S3). The O serogroup of 175 (73.5%) isolates was identified (according to criteria of >85% nucleotide similarity level across at least 60% target sequence) from WGS data using SeroTypeFinder (17); the remaining 63 (26.5%) isolates were unassignable (O serogroup untypeable). Unassignable serogroups were identified across most phylogroups (A, 6 of 25, 24.0%; B1, 36 of 126, 28.6%; B2, 7 of 30, 23.3%; D, 1 of 22, 4.5%; E, 2 of 9, 22.2%) and non-E. coli species (11 of 23, 47.8%). Preliminary SNP analysis of core genes revealed that seven phylogroup B2 gST535 isolates were very similar (<10 SNPs) and were identified in the same locale (sites 3 and 4). Additional core genome SNP analysis and pairwise SNP comparisons of WGS data from the same seven gST535 isolates were undertaken to provide enhanced phylogenetic resolution using one of the seven target isolates as a reference sequence (Fig. 3). At sites 3 and 4, six of the gST535 isolates recovered from water and fecal samples of multiple wildlife hosts (rat, possum, and avian), between November 2017 and May 2018, were at least 99.68% similar at the core genome SNP level (total SNPs, 1,108). These six gST535 isolates obtained from sites 3 and 4 differed only by 1 to 6 SNPs (Fig. 3), indicating clonal isolates. Similarly, clonal isolates representing gST161, gST251, and gST395 were isolated from sites 2, 3, 4, and 5 during separate visits and from different sample types (Fig. 3).

### Examination of the *Escherichia* virulome.

**FIG 4:**
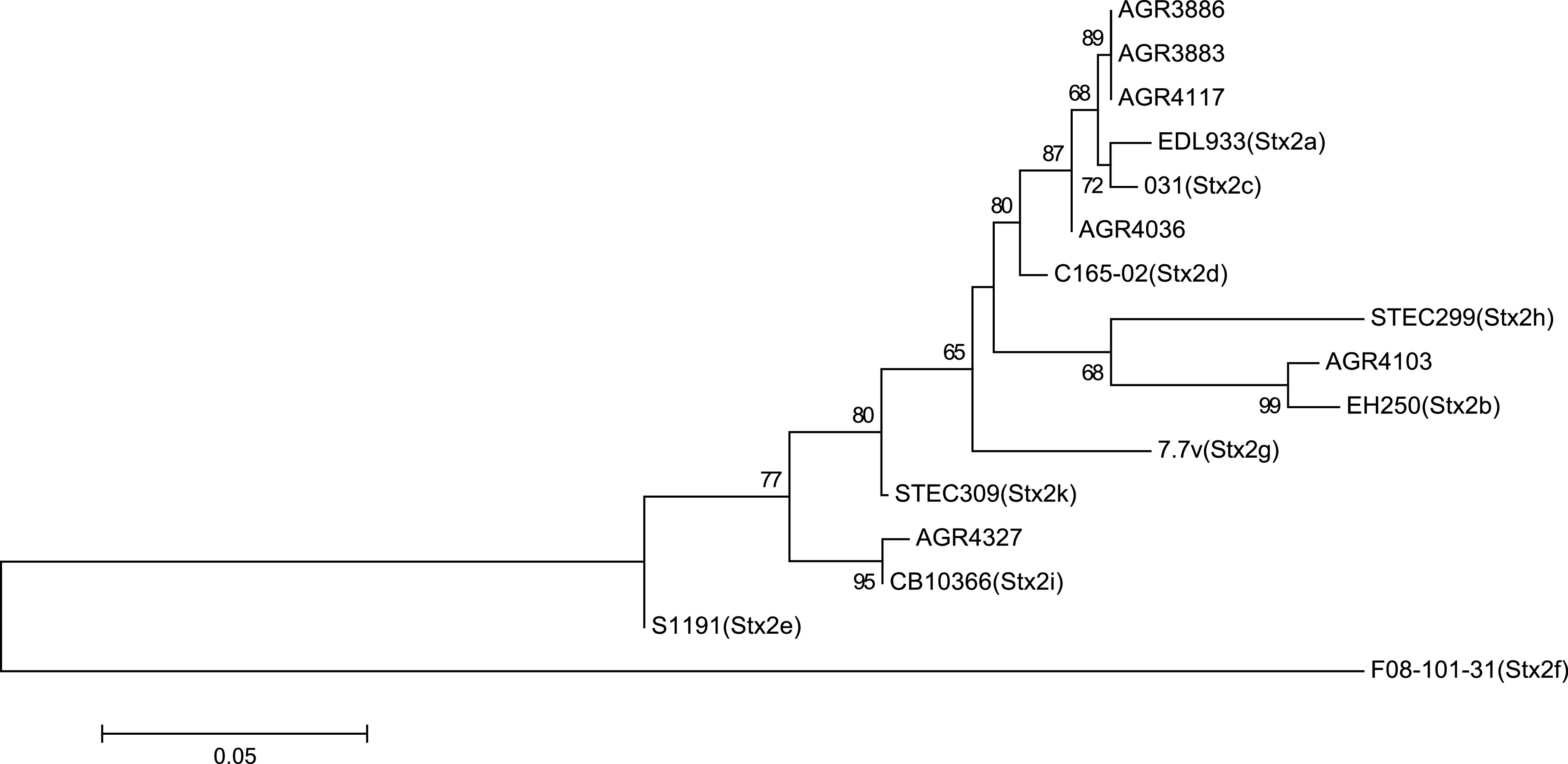
Phylogenetic tree of Stx2 subtypes by the neighbor-joining method. The neighbor-joining tree containing AGR isolate designations from this study was inferred from comparison with combined A and B holotoxin amino acid sequences of all Stx2 subtypes. Numbers on the tree indicate bootstrap values calculated for 1,000 subsets for branch points of >50%. Bar, 0.05 substitutions per site. Stx_2a_, EDL933 (X07865); Stx_2b_, EH250 (AF043627); Stx_2c_, 031 (L11079); Stx_2d_, C165-02 (DQ059012); Stx_2e_, S1191 (M21534); Stx_2f_, F08-101-31 (AB472687); Stx_2g_, 7v (AY286000); Stx_2h_, STEC299 (CP022279); Stx_2i_, CB10366 (FN252457); Stx_2k_, STEC309 (CP041435).

Although no E. coli isolates were both *stx* and *eae* positive, 10 E. coli isolates (1.41%) were *stx*_2_ positive and 19 E. coli (2.7%) isolates were *eae* positive, indicative of the atypical enteropathogenic E. coli (aEPEC) pathotype. The *eae* gene encodes a protein, intimin, that is involved in the intimate attachment of bacteria to human epithelial cells (18). Fifteen *eae*-positive E. coli isolates, representing A, B1, B2, and F phylogroups from 15 separate samples, obtained from sites 1, 3, 4, and 5, underwent WGS analysis and further *eae* subtyping using BLAST. Seven distinct *eae* subtypes, alpha2 (*n* = 2), beta, beta2 (*n* = 4), epsilon2, kappa, iota, and theta (*n* = 5), were identified from possum feces, avian feces (*n* = 4), sediment (*n* = 3), and water (*n* = 7) (Table S3). Similarly, five E. coli isolates (four phylogroup B1 and a single B2) from bovine feces, water, soil, and periphyton samples positive for the *stx*_2_ gene using RT-PCR underwent WGS and *stx*_2_ subtyping (Table S3). *stx*_2_ genes from serotype O nontypeable:H20 (AGR3883, AGR3886, and AGR4117) were most similar to *stx*_2c_, matching an uncommon *stx*_2_ variant (accession no. FM998860) (Fig. 4). AGR4036 (O9:H21) was also most similar to *stx*_2c_ (serotype O91, accession no. CP015244), and AGR4103 (O33:H6) was most similar to *stx*_2b_ (CP027586, 99.9% nucleotide match) (Fig. 4). Another isolate, AGR4327 (O30:H25), not identified as *stx*_2_-positive using RT-PCR due to primer mismatches, possessed an *stx*_2_ variant most similar to a newly described toxin variant, *stx*_2i_ (ONT:NM, *Stx*_2_ A-subunit 99.5% nucleotide match, accession FN252457) (Fig. 4), originally isolated from raw milk (19). Other STEC virulence factors were rare (Table S3) and included *esp*P (serine protease, *n* = 2) (20), *ehx*A (enterohemolysin, *n* = 2) (21), *iha* (*Irg*A homologue adhesin, *n* = 4) (22), *etp* (type II secretion system, *n* = 1) (23), and the *ter*ZABCDE operon (tellurite gene resistance operon, *n* = 7) (24), but only eight (50%) of the additional STEC virulence factors were identified in STEC isolates, four (25%) in aEPEC isolates, and the remaining four (25%) in WGS data from neither STEC nor aEPEC. None of the *stx*- or *eae*-positive E. coli isolates possessed any antimicrobial resistance genes. Two aEPEC isolates (*eae* beta2) and a single STEC isolate (*stx*_2b_) were recovered from the native bush sampling site, and a further two STEC isolates (*stx*_2c_) were recovered from opportunistic sampling of bovine feces at site 1 but outside the reserve perimeter fence (see supplemental material).

**FIG 5:**
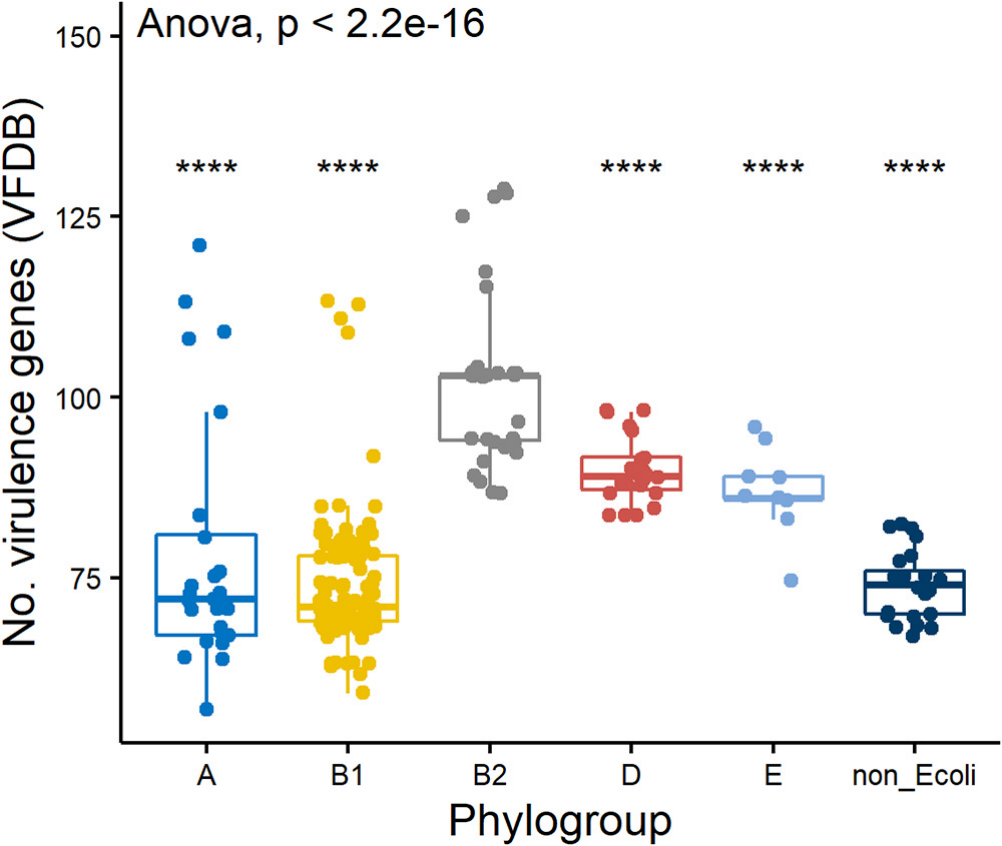
Box and whisker plot of total virulence genes identified using the virulence factor database identified from WGS data of E. coli (*n* = 212) of phylogenetic groups A, B1, B2, D, and E and non-E. coli represented by Escherichia marmotae (*n* = 22) and *E. ruysiae* (*n* = 1). The boxes show median values and span lower to upper quartiles, the whiskers show the highest and lowest values within 1.5 times the interquartile range, and dots beyond the whiskers show potential outliers. A Student’s *t* test of variance was used to compare numbers of virulence genes from phylogroup B2 with each other phylogroup and non-E. coli group; all pairwise comparisons were *P* < 0.0001. A one-way analysis of variance (ANOVA) was used to compare counts from all phylogroups and non-E. coli group.

**FIG 6:**
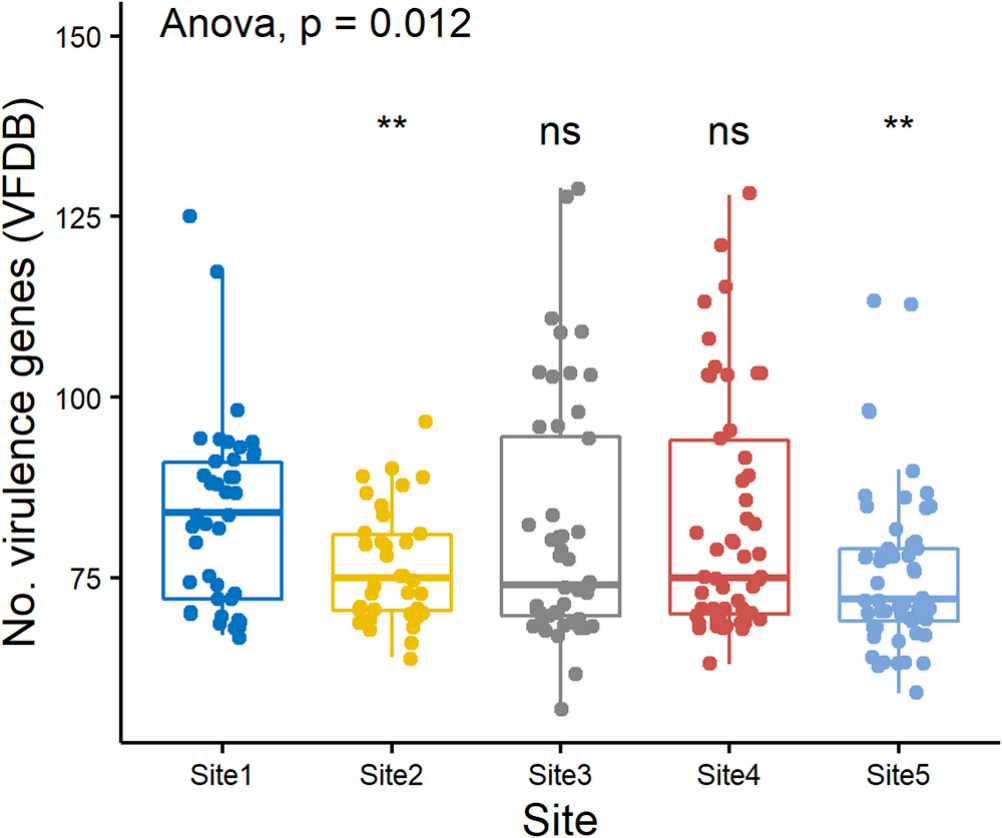
Box and whisker plot of total virulence genes identified using the virulence factor database identified from WGS data of E. coli (*n* = 215) and non-E. coli represented by Escherichia marmotae (*n* = 22) and *E. ruysiae* (*n* = 1) from each of five field sample sites. The boxes show median values and span lower to upper quartiles, the whiskers show the highest and lowest values within 1.5 times the interquartile range, and dots beyond the whiskers show potential outliers. A Student’s *t* test was used to compare numbers of virulence genes from site 1 with each other site; significant pairwise comparisons (**) were *P* < 0.01, and ns was not significant, *P* > 0.05. A one-way ANOVA was used to compare counts from all five sites.

In total, 259 and 58 different virulence factors were identified from WGS data using VFDB (25) and VirulenceFinder (26, 27), respectively. Using VFDB data, the 15 *eae*-positive E. coli isolates possessed the highest number of virulence genes per isolate (mean = 118, SD = 7.4) due to the presence of locus for enterocyte effacement (LEE) translocator (e.g., *esp*A, *esp*B, *esp*D, *tir*), type III secretion system (*esc*), and non-LEE effector (*nle*A, *nle*B, *nle*E) alleles. Non-E. coli (*E*. *marmotae* and *E. ruysiae*) isolates had the fewest virulence genes (mean = 74, SD = 4.8), and isolates from phylogroups B2 (102.3, 12.2), D (89.9, 4.4), E (87.1, 5.8), and A (78.4, 16.9) had higher numbers of virulence genes than most B1 isolates (74.3, 8.8) (Fig. 5). Although isolates for WGS were not selected randomly, more virulence genes were identified from phylogroup B2 isolates than from other phylogroups (Fig. 5). Additionally, there was a correlation between number of virulence genes per isolate and site (*P* = 0.012, ANOVA), likely influenced by phylogroup B2 virulome data; VFDB data from isolates recovered from site 2 (only a single phylogroup B2) and site 5 (phylogroup B2 absent) indicated fewer virulence genes per isolate (*P* < 0.01, pairwise *t* tests) than those recovered from site 1 (13 phylogroup B2), which had the highest median number of virulence genes (Fig. 6). No statistically significant variation in numbers of virulence genes was observed for sites 3 and 4. The distribution of 13 representative virulence genes identified using VirulenceFinder (26, 27) from WGS data and present in at least 14 of the 238 WGS isolates across sites and phylogroups was investigated (Table 4). The most abundant virulence factors identified were *iss* (153 isolates, 64.3%), *lpf*A (136, 57.1%), *tra*T (135, 56.7%), and *gad* (101, 42.4%). Examination of this subset of virulence factors demonstrated a remarkable similarity of allele distribution between isolates of the same phylogroup (Table 4). For example, *lpf*A, *iss*, *gad*, and *tra*T were widespread in the virulome of phylogroup B1. *fyu*A and *irp*2 were overrepresented in phylogroup B2, as well as *pic* and *vat*, genes encoding serine protease autotransporters of *Enterobacteriaceae* (SPATEs) (Table 4). Three SPATE genes were detected: *esp*P from 2 STEC isolates (AGR4036 and AGR4117), *pic* from 16 E. coli isolates (4 phylogroup B1, 12 phylogroup B2), and *vat* from 14 phylogroup B2 isolates. Finally, *eil*A and *air* were found only in phylogroups D and E (Table 4). Interestingly, neither of the two most common alleles, *iss* and *lpf*A, was found in *E. marmotae* and the single *E. ruysiae* isolate.

**TABLE 4:** Prevalence of virulence genes among E. coli phylogroups and non-E. coli species (*E. marmotae*, *n* = 22; *E. ruysiae*, *n* = 1) isolated from different environmental sample types

Isolation group or source (*n*)	% of isolates carrying the indicated virulence gene^*a*^												
	*traT*	*iss*	*lpfA*	*gad*	*astA*	*eilA*	*air*	*vat*	*pic*	*irp2*	*fyuA*	*iroN*	*kpsMII*
Phylogroup													
A (25)	24.0	52.0	4.0	32.0	12.0	0	0	0	0	8.0	12.0	4.0	4.0
B1 (126)	73.8	80.2	98.4*	38.1	21.4	0	0	0	3.2	4.8	4.8	0.8	0
B2 (30)	56.7	70	0	43.3	10	0	0	46.7*	40*	93.3*	93.3*	43.3*	36.7
D (22)	59.1	59.1	36.4	72.7	13.6	100	72.7	0	0	0	0	13.6	72.7
E (9)	22.2	22.2	0	55.6	11.1	88.9	88.9	0	0	0	0	0	0
Non-E. coli (23)	13.0	0	0	47.8	56.5	0	0	0	0	0	0	0	82.6
Sample type													
Feces (68)	57.4	66.2	55.9	45.6	17.6	16.2	11.8	8.8	7.4	19.1	19.1	11.8	16.2
Avian (23)	52.2	52.2	39.1	43.5	21.7	17.4	13.0	13.0	8.7	26.1	26.1	17.4	21.7
Nonavian (45)	60.0	73.3	64.4	46.7	15.6	15.6	11.1	6.7	6.7	15.6	15.6	8.9	13.3
Periphyton (35)	48.6	68.6	45.7	51.4	11.4	11.4	8.6	8.6	5.7	28.6	28.6	14.3	25.7
Sediment (37)	51.4	59.5	54.1	45.9	21.6	13.5	8.1	5.4	5.4	5.4	8.1	0	24.3
Soil (23)	73.9	69.6	78.3	34.8	30.4	8.7	8.7	4.3	4.3	8.7	8.7	4.3	17.4
Water (75)	57.3	61.3	58.7	36.0	26.7	10.7	10.7	2.7	8.0	14.7	14.7	6.7	18.7

a Virulence genes in each strain that underwent WGS (238) were identified using VirulenceFinder using default parameters. Numbers listed represent the percentage of isolates that carry the designated allele: *traT*, outer membrane protein complement resistance; *iss*, increased serum survival; *lpfA*, long polar fimbriae; *gad*, glutamate decarboxylase; *astA*, enteroaggregative E. coli heat-stable toxin 1; *eilA*, Hil*A*-like regulator; *air*, enteroaggregative E. coli immunoglobulin repeat protein; *vat*, vacuolating autotransporter protein; *pic*, protein involved in colonization; *irp2*, high molecular weight protein 2, nonribosomal peptide synthetase; *fyuA*, siderophore receptor; *iro*N, novel catecholate siderophore receptor; *kpsMII*, polysialic acid transport protein. Statistical significance (*, *P* < 0.05) for each gene was determined using Fisher exact tests between two chosen groups: for phylogenetic groups, comparisons were made between the group with the highest prevalence and the group with the next highest rate of prevalence for that particular gene; for sample type, comparisons were made between the group of feces isolates and the group of nonfeces isolates.

### Detection of AMR determinants.

Antimicrobial resistance (AMR) gene detection was rare with only one isolate, AGR3730, displaying a multidrug resistance genotype of plasmid-borne aminoglycoside, beta-lactam, and sulfonamide resistance (Table S3). Fosfomycin resistance, encoded by the chromosomal *fos*A7 gene, was associated with 7 E. coli isolates (1 from bovine feces, 5 from water, soil, or sediment samples from third or fourth order streams, and the last from wetland [site 5], which all had agricultural influences), which were ST-75 (*n* = 3) and ST-3234 (*n* = 4) (Table S3) and matched the genotype *fos*A7.5 variant recently described in E. coli isolated from patients in Canadian Hospitals (28). AGR3730 displayed a plasmid-associated *bla*_TEM-1C_ *str*A*-str*B′ *sul2* multidrug resistance genotype virtually identical to that of the IncI complex plasmid pSCU-123-2 (99.96% identity at the nucleotide level, CP051718) (29) and was resistant to ampicillin and streptomycin but sensitive to cotrimoxazole. Antibiotic sensitivity testing of the seven *fos*A7-positive E. coli isolates showed a resistant phenotype.

## DISCUSSION

E. coli is a common inhabitant of the mammal gut microbiome and has been extensively characterized in studies that focus on human and veterinary clinical isolates or those from urban or pastoral sampling sites. Characteristics of E. coli recovered from temperate mammal-free environments are unknown. New Zealand temperate environments are unique in that in their native state they are virtually free of terrestrial mammals, are minimally influenced by any negative man-made activities, and thus represent sites for the study of E. coli where high levels of endemic biodiversity occur. This study aimed to identify virulence factors associated with environmental E. coli, including STEC, and examine their prevalence, to improve the assessment of public health risk from recreational exposure. Five freshwater field sampling sites were selected that provided a range of observed land-use impacts to examine the complexity and variation of E. coli at such locations. Site 1 is an extensively pest-managed native forest site with high endemic biodiversity, negligible levels of landscape modification, and a low biomass of introduced plant and animal species; sites 2, 3, and 4 are sites impacted by pastoral sheep and beef farming and low endemic biodiversity, and site 5 is a constructed wetland site treating tile drainage on a dairy farm with very low levels of endemic biodiversity (Table 1).

Despite the active management of introduced predator species at site 1 and an absence of native and introduced waterfowl at this sample site, E. coli was still present in freshwater with concentrations ranging from 4 to 133 MPN/100 mL water (Fig. 1) with the maximum concentration associated with a recent prior heavy rainfall event. While the original source of these E. coli isolates is unknown, increased E. coli concentrations may be due to mobilization of bacteria from sediment or periphyton during rapid flow events (30, 31). In contrast, E. coli counts in water samples for site 5 were highly variable between visits (0 to 7,710 MPN per 100 mL) (Fig. 1). This is possibly due to reduced hydraulic retention time within the wetland during heavy rainfall events and increased flow velocities mobilizing bacteria from storage reservoirs within the wetland.

Understanding the risk to public health from recreational exposure to zoonoses at freshwater sites and the associated prevalence of pathogens is important in reducing human disease. New Zealand waterways are considered unsuitable for recreation if a single water sample contains greater than 540 E. coli cells per 100 mL water (2). Several water samples from the higher order freshwater sites (Fig. 1, Table 1) flowing through agricultural landscapes had increased concentrations of E. coli, exceeding these guidelines and, in parallel, an increased presence of pathogenic E. coli with *stx*_1_, *stx*_2_, or *eae* alleles which after heavy rainfall events and overland-flow transport could lead to increased public health risks. Although E. coli was identified more commonly in greater numbers in sediment samples than in soil, the sediment counts were typically lower than water by at least an order of magnitude. Nevertheless, stream sediments are a potential source of stored E. coli that could be entrained during elevated stream flow (30, 32, 33).

STEC is a common inhabitant of the ruminant gut, and *stx*-positive cattle are present on most New Zealand farms (34). STEC isolates were recovered rarely (4%, 1 of 25) from the opportunistic bovine fecal samples, but *stx* genes were identified more frequently in sample enrichments from bovine feces (44%, 11 of 25) using RT-PCR. Several of the fecal samples were from aged (more than 7 days) material, influencing recovery and indicating potentially reduced survival and competitiveness of STEC compared to those of non-STEC in subsequent fecal culture enrichments for isolation purposes. The use of environmental culture enrichments and boiled lysates as a source of template DNA for RT-PCR provides an indication of the presence or absence of a particular target gene with low limits of detection (<10^2^ CFU per mL) (34) but precludes the absolute quantification of target amplicons when using direct PCR of purified DNA extracts from environmental samples. The identification of STEC and intestinal pathogen virulence factors from freshwater samples and potential impact of recreational exposure is often more challenging than epidemiological analysis of terrestrial samples (e.g., feces and soil) due to the diffuse and temporal nature of pathogens in samples obtained from freshwater environments and the need for concentration by filtration prior to enrichment. Nevertheless, 68 of 189 (36.0%) environmental sample enrichments were *stx*_1_ and/or *stx*_2_ positive, and STEC isolates were isolated from the native bush site, despite the absence of most mammals, in addition to the other four field sampling sites impacted by pastoral farming. Overseas, small outbreaks of STEC infection involving a few infected individuals have been identified from recreational exposure during swimming in lakes (9, 10). STEC isolates were also recovered in low numbers from freshwater rivers in France (0.9%, 6/651) (11), Argentina (4 *stx*_2_-positive STEC isolates) (12), and Poland (7.3%, 14/192) (13). Previous work in New Zealand has suggested that contact with recreational waters is a significant environmental risk factor associated with human STEC infections (8), but little work has been undertaken to identify *stx*-positive isolates from freshwater samples in catchments with contrasting land use. A recent New Zealand study that investigated 52 water samples from 16 separate freshwater sites with dairy, urban, or sheep and beef observed land uses, with historically high levels of generic E. coli, detected STEC in 13 water samples from 11 rivers (<0.25 MPN/100 mL) (35). Direct PCR of DNA extracts from water samples detected the *stx*_1_ gene only in three samples (5.8%), *stx*_1_ and *stx*_2_ in four samples (7.7%), and *stx*_2_ only in six samples (11.5%). Only one STEC isolate (O177:H25) was recovered.

RT-PCR of purified isolates was used to identify those positive for *stx*_1_ and/or *stx*_2_ and the outer membrane protein *eae* recovered from feces and environmental sources. None of the *stx*_2_ genes identified from isolates were clinically significant *stx*_2a_ or *stx*_2d_ variants (36). Four of the STEC isolates from feces, soil, and water possessed *stx*_2_ variants most similar to *stx*_2c_, rarely associated with severe disease, and another *stx*_2_ allele from an isolate recovered from endemic native forest (site 1) matched *stx*_2b_ from an E. coli isolate recovered from water (Fig. 4). The final *stx*_2_ variant was recovered from an E. coli isolate isolated from periphyton with a toxin type most similar to that of *stx*_2i_, whose toxin product has yet to be characterized but has not been associated with clinical disease and has been recovered only from raw milk (19) and bivalves (37). Other STEC-associated virulence factors (*esp*P, *etp*D, *ehx*A, *iha*, and *ter*ZABCDE) were uncommon and were often identified in isolates that were *stx* or *eae* positive (Table S3). E. coli isolates with *eae* subtypes alpha2 or beta2 were isolated from freshwater samples at the native forest site 1, but none were isolated downstream from the pastoral site 2. In contrast, *eae*-positive E. coli isolates were isolated from wildlife feces (avian and possum) and freshwater samples from sites 3, 4, and 5, where wildfowl species such as mallard (Anas platyrhynchos) and the native pūkeko (Porphyrio porphyrio melanotus) were common in the more open pastoral landscape. Many animal species (38), including wildfowl (39), may be colonized by *eae*-positive E. coli with recovery also from environmental samples (40, 41), including from macrophytic green algal mats (*Cladophora*) (42). There was no evidence from New Zealand surveillance data encompassing 2016 to 2020 (43) that the *stx*- or *eae*-positive E. coli serotypes isolated in this study were of clinical importance. However, the prevalence with which *stx* genes were identified from environmental sample enrichments in our study, especially from locales associated with agriculture, is a further indication of potential risk associated with recreational exposure to water (11). Furthermore, 26.5% of the E. coli isolates that underwent WGS as part of this study did not match O-antigen biosynthesis genes included as part of SeroTypeFinder (17), which suggests that they are unlikely to be established clinical serogroups.

To provide bacterial subtyping and refinement of the isolate collection for WGS, isolates were differentiated by Sanger sequencing of a 284-bp region of the hypervariable *gnd* allele that encodes 6-phosphogluconate dehydrogenase (44). Due to its close proximity to the O-antigen biosynthesis gene cluster, *gnd* is often affected by homologous recombination that influences O serogroup, and to date, over 600 different 284-bp partial *gnd* sequence types have been recognized (45). This subtyping method enabled presumptive clonal isolates to be readily identified and considered for subsequent WGS analysis. Core genome SNP analysis of WGS data from this study provided a clear example of the broad diversity of fecal E. coli phylogroups and newly described *Escherichia* species, *E. marmotae* and *E. ruysiae*, recovered from diverse samples and catchment land use. The panel of 238 isolates that underwent WGS analysis were not selected randomly, but it is noteworthy that most phylogroups (A, B1, B2, D, and E) (Fig. 2, Table S3) were identified from all five sites, except phylogroup B2, which was absent from the WGS isolate panel from the dairy farm wetland, site 5. The apparent scarcity of phylogroup B2 isolates at the dairy wetland (site 5) maybe due to a greater proportion of phylogroup B1 isolates originating from bovine feces. Despite the abundance of phylogroup B2 at site 1, the site with limited human access, the use of VirulenceFinder and VFDB indicated that B2 isolates have an increased prevalence of extraintestinal virulence genes (Table 4) and virulence genes overall (Fig. 5). Previous studies have noted that, in general, phylogroup B1 strains are the dominant E. coli strains of the animal microbiota (46), and the presence of dairy cattle and the local New Zealand dairy farm practice of spraying effluent onto pastures as a source of nitrogen may influence the predominance of phylogroup B1 and apparent lack of B2 from this wetland site. In a previous study, phylogroup B1, as well as E, was observed to be more abundant in samples taken from pastoral sites, whereas phylogroup B2 was more abundant in those from forested sites (11). Other studies have described phylogroup B1 strains as generalists linked to contrasting conditions and aquatic vegetation and absent from urban areas (47).

A study that included the analysis of human, avian, wildlife, companion animals, and water E. coli isolates noted that B2 isolates were more abundant from humans than phylogroup B1 isolates (46). Extraintestinal pathogenic E. coli (ExPEC) is a subset of E. coli that is predominantly phylogroup B2 and is commonly the causative agent of infection during entry to normally extraintestinal sites such as the urinary tract (48, 49); specialized virulence factors (siderophores, toxins, and adhesins) are thought to mediate infection upon entry to such sites (27). Previous studies have identified phylogroup B2 ExPEC strains of public health relevance from diverse environmental samples, including freshwater sites (46, 48), and it is noteworthy that both phylogroup B2 isolates (Fig. 5) and genes annotated as virulence factors (Fig. 6) were present more frequently in isolates from the native forest, site 1, with high levels of endemic biodiversity, indicating a possible role of some ExPEC virulence factors in environmental survival.

Despite many studies involving the characterization of E. coli from humans and pastoral animals, the comparatively recent discovery of *E. marmotae* (cryptic clade V) (50) and *E. ruysiae* (cryptic clades III and IV) (51) suggests that they are nonpathogenic (52, 53) and an uncommon component of the human or livestock gastrointestinal tract, despite the progenitor type strains of both newly described species being obtained from feces: *E. marmotae* HT073016^T^ obtained from the feces of wild rodents in Qinghai-Tibet plateau (50, 54) and *E. ruysiae* NCTC 14359^T^ obtained from the feces of a healthy human on a single occasion (51).

Previous studies have also demonstrated that *E. marmotae* is associated with fecal samples from avian species (14, 55). However, in this study, *E. marmotae* and *E. ruysiae* were identified only in a single avian fecal sample (4.3%, 1 of 23) and were isolated more commonly from nonfecal samples (95.2%, 20 of 21, *P* < 0.05, Fisher’s exact test), especially those obtained from freshwater environments, such as periphyton, sediment, and water samples. *E. marmotae* have also been isolated from mammals, but no long-term colonization or clinical disease has been observed (50, 52, 56, 57), implying that the mammalian gastrointestinal tract is unlikely to be the primary colonization site for *E. marmotae*.

The role of wildlife and birds in the environmental spread of E. coli is poorly understood (58), but our high-resolution phylogenetic analysis shows at an unprecedented scale that some gST clones were shared between wildlife and the environment (Fig. 3). Closely related isolates (<5 SNPs) were present within the same general geographical area (e.g., within sites 3 and 4 and within site 5) over several months and recovered from environmental and wildlife or livestock fecal samples. For example, clonal isolates of gST535 were widespread, isolated from water, possum, rat, and avian feces from sites 3 and 4. E. coli gST535/ST681 have been recorded previously only from wild boar in Europe (59) and primates in the Gambia (60). Such clones (<5 SNPs) in this study were isolated only from particular geographical areas, so whether survival and persistence in the environment or local wildlife contribute to the amplification and maintenance of E. coli within distinct environmental sites, or if wildlife is a spillover host of certain bacteria (61), requires further study.

New Zealand is one of the three lowest users of antibiotics to treat livestock animals in the OECD (62), but antibiotic use is common in the dairy industry to prevent and treat mastitis (63). Nevertheless, levels of overall AMR (3.36%, 8 of 238) and multidrug resistance as indicated by WGS of E. coli were low (0.42%, 1 of 238) in this study compared to those in similar studies undertaken overseas where the prevalence of multidrug-resistant E. coli was 14% to 30% (11, 64). A more reflective impact of human and agricultural activities on AMR prevalence than that in this study may be established from analysis of freshwater samples obtained from catchments with higher urban population densities, inflow from additional human wastewater discharge sites, and/or runoff from agricultural land with increased animal density.

Few studies have examined E. coli recovered from environmental sites of contrasting land use for the purposes of establishing public health risk (11). Because of the almost complete absence of endemic terrestrial land mammal species (except the long-tailed bat, Chalinolobus tuberculatus, and the lesser short-tailed bat, Mystacina tuberculata), New Zealand habitats offer a unique opportunity to investigate the complexity and variability of E. coli populations from environmental sites of contrasting land use. This is the first study to take a longitudinal sampling approach to examine baseline levels of the E. coli virulome using WGS data from freshwater catchments and sample environments with pastoral activity and other contrasting sample sites where an absence of pastoral activity is strictly enforced, endemic species are actively protected, and human access is minimal. Although only one native forest site (site 1) was sampled, the overall abundance of E. coli phylogroup B2 possessing a virulome more extensive than that of other sites and E. coli phylogroups was not anticipated. Further studies are required including additional native forest and matching pastoral/urban sites to investigate whether the findings from this study can be translated to other sites.

This study revealed the extreme complexity and variability of E. coli recovered from environmental sites of contrasting human activity, and further work is required to understand the public health implications of transmission in these locations so that meaningful reductions can be achieved. Antimicrobial-resistant E. coli and isolates with clinically relevant virulence factors such as *stx*_1_, *stx*_2,_ and *eae* associated with human intestinal disease were rare, but *stx* and *eae* prevalence in enrichments of environmental samples was more common. This indicates that the presence of pathogenic E. coli, including STEC, may be more common than previously considered, but these E. coli types are present in low abundances and may possess genetic characteristics less commonly associated with human disease. Virulence factors associated with ExPEC were also identified in environmental E. coli isolates, especially those from phylogroups B2 and D, and newly described *Escherichia* species and suggest an additional role in environmental survival and persistence. Our systematic spatiotemporal surveillance of sites and analysis of WGS data were also able to clearly demonstrate the clonality of E. coli isolates from wildlife and the environment. These observations indicate that there could be spillover from wildlife to the environment or transmission to wildlife from the environment. Further investigation is required to understand the prevalence of environmental E. coli types and the newly described *Escherichia* species in wildlife and mammal-free environments using high-resolution methods such as WGS. This would assist in understanding their relevance to water quality and public health risk assessments, particularly where these *Escherichia* types may be contributing to poor water quality.

## MATERIALS AND METHODS

### Sample site details.

Five field sites (Table 1) in the North Island of New Zealand were sampled on five or six occasions over 11 months (August 2017 to June 2018). Four of the five sites were from the Manawatū River catchment. Site 1 is a headwater stream site within Pūkaha Mount Bruce, an intensively managed 942-ha conservation reserve where trapping of introduced predatory species (mustelids, possums, hedgehogs, and rodents) is undertaken to enhance native biodiversity. This sample site is free of endemic and introduced waterfowl species. Site 2, a fourth-order freshwater site at Hamua Bridge (Mākākahi River, Tararua) where sheep and beef farming operations form much of the adjacent land use, was 21.5 km (direct distance) downstream from site 1.

Sites 3 and 4 were nearby the confluence of the Mangaterā and Mākirikiri streams (Tararua), south of Dannevirke (population 5,200). Livestock farming (sheep, beef, dairy) also dominated the catchment land use for both these streams. Both streams were sampled; the sampling site on the Mākirikiri stream (site 3) was immediately downstream from a small stand of native forest and 30 m upstream of the confluence with the Mangaterā stream. The sampling site on the Mangaterā stream (site 4) was about 50 m upstream of the confluence with the Mākirikiri stream.

Samples were also obtained from the inflow, middle, and outlet of a constructed wetland (site 5) in the Toenepi River catchment in the Waikato, a major dairying region (containing 22.7% and 1.13 million cows) of New Zealand (65). The wetland intercepts and treats subsurface tile drainage waters from intensively grazed dairy pasture (66).

### Environmental sampling and bacterial recovery.

At each site, water, sediment (not site 2), soil, periphyton, and fecal samples were collected. E. coli cells were enumerated from water samples (100 mL) using Colilert-18 and Quanti-Tray/2000 (IDEXX, ME, USA) incubated at 35°C (18 to 21 h) (67) for the recovery of stressed cells to determine the most probable number of E. coli cells per 100 mL (MPN/100 mL). E. coli colonies were also recovered by filtering water samples (100 mL) and soil and sediment extractions through 0.45-μm nitrocellulose filters using positive pressure and incubating the filter on CHROMagar ECC plates (CHROMagar Microbiology, Paris, France). Water samples were also filtered as described above with the filter enriched in 10 mL EC broth incubated at 35°C (18 to 21 h).

Freshwater sediment samples were obtained using a stainless-steel shovel and sieved through a mesh of approximately 3 mm to retain coarse particles. Adherent bacteria were removed from the sieved sediment (10 g) using 90 mL peptone (0.1%) saline (0.85%) solution (30) and shaken gently by hand for 1 min. The material was left for 10 min to settle before E. coli isolates were enumerated in the supernatant using Colilert-18 and Quanti-Tray/2000. Sediment material (1 g) was also enriched in 9 mL EC broth by incubating at 35°C (18 to 21 h) followed by subculturing onto CHROMagar ECC plates.

Soil sample sites were collected 5 to 10 m from freshwater sample sites. A composite soil sample from each site (~70 g) was obtained using a sterile 150-mm stainless steel corer and processed for the enumeration and recovery of E. coli as described previously for sediment samples, except a 1:10 dilution of gelatin (1% [wt/vol], pH 10.3) preparation diluted in (NH_4_)_2_HPO_4_ (0.1 M) extractant was used (68).

The dry weight (105°C for 24 h) of sediment and soil samples was determined to establish the MPN of E. coli present per gram dry weight of soil/sediment using Quanti-Tray/2000 data.

We obtained periphyton samples from all stream sites by carefully removing a fully submerged rock from the waterway and wiping an area of approximately 100 cm^2^ using a sterile sponge swab (EZ-Reach Sponge Sampler, World Bioproducts, WA, USA). At the wetland (site 5), where rocks were absent, biofilm samples were obtained by wiping submerged vegetative material. The sterile sponge swab was stomached for 1 min with 25 mL EC broth (Oxoid, Hampshire, UK) and incubated at 35°C (18 to 21 h). Broth culture (50 μL) was inoculated onto CHROMagar ECC plates, streaked for individual colonies, and incubated at 35°C (18 to 21 h).

Opportunistic fecal material was obtained using a sterile Amies swab (Copan Diagnostics Inc., Brescia, Italy) or sterile specimen container with scoop cap, diluted 1:100 in EC broth, and incubated on CHROMagar ECC plates for isolation of individual colonies as before. Bovine and ovine fecal enrichments were inoculated with a composite sample obtained by combining fecal material recovered from 3 well-separated fecal deposits, where available.

For all sample types, generally the growths from four separate subcultured colonies from each sample preparation was stored at −80°C by resuspending individual colonies in EC broth containing glycerol (33% [wt/vol]). Crude boiled DNA isolate and enrichment extracts from all samples were performed and stored at −20°C.

### Molecular analyses and subtyping of isolates.

A 284-bp partial *gnd* sequence was amplified by PCR (2*gnd*F and 2*gnd*R primers) and Sanger sequenced (ABI3730 DNA analyzer, Massey Genome Service, Massey University, Palmerston North, New Zealand) as described previously (44). The *gnd* sequence type (gST) from each E. coli isolate was identified using a custom-made *gnd*Db database (45) of 614 distinct 284-bp *gnd* sequences obtained from genome sequence data.

Real-time PCR (RT-PCR) was used to determine the prevalence of the E. coli virulence factors responsible for the expression of Shiga toxin 1 (*stx*_1_), Shiga toxin 2 (*stx*_2_), and an epithelial cell adherence factor, intimin (*eae*). Individual isolates were examined by using a crude boiled lysate from a single colony as a source of DNA template; similarly, environmental sample enrichments were examined for the presence/absence of each virulence factor using a washed boiled preparation of postenrichment samples. Primers and probes specific for *uid*A (β-glucuronidase) were also included in RT-PCRs for the putative identification of E. coli. RT-PCR methods, primers, and probes have been described previously (69). PerfeCTa MultiPlex qPCR ToughMix (Quanta Biosciences, Gaithersburg, MD, USA) was included in the reaction mix, and amplification was carried out using a Rotor-Gene Q RT-PCR cycler (Qiagen). Culture enrichment samples and individual isolates were deemed positive for the RT-PCR target with a *C*_q_ (quantification cycle) of ≤35.

Escherichia marmotae (cryptic clade V) and *E. ruysiae* (cryptic clade IV) were identified using the PCR primers and conditions described previously (14).

### Antimicrobial sensitivity testing of isolates.

Antimicrobial sensitivity testing was undertaken on E. coli possessing AMR genes to determine antibiotic resistance phenotype using the Kirby-Bauer disk diffusion method according to EUCAST standard methods (70). E. coli NZRM916 (ATCC 25922, DSM1103, NCTC12241), a recommended reference strain for aerobic antimicrobial sensitivity testing, was included in all disk diffusion experiments as a negative control.

### Whole-genome sequencing and phylogenetic analysis of isolates.

DNA extractions and library preparations for WGS were undertaken as described previously (34) using the QIAamp DNA minikit (Qiagen, Hilden, Germany), and libraries were prepared using the Nextera XT DNA library preparation kit (Illumina, San Diego, CA). WGS was undertaken by Novogene Limited (Beijing, China) using the Illumina HiSeq paired-end v4 platform (2 by 125 bp). The Nullarbor2 pipeline (71) was used including read trimming of adapters to process and examine WGS read data for *de novo* genome assembly using SKESA (v.2.2.1) (72), annotation using Prokka (v.1.13.3) (73), and phylogenetic analysis using Snippy (v.4.2.1) (74). Randomized Axelerated Maximum Likelihood (RAxML) next-generation (75) maximum-likelihood trees were generated of the core SNP alignment using a general time-reversible model and random seed to perform 20 tree searches using 10 random and 10 parsimony-based starting trees. The best-scoring maximum-likelihood tree was viewed in SplitsTree (v.4.14.8) (76). The *Stx*_2_ holotoxin amino acid sequence was generated from the joined *Stx*_2A_ and *Stx*_2B_ subunits and aligned to determine the genetic distances. Phylogenetic analysis and reconstruction of phylogenetic trees were undertaken with the maximum-likelihood algorithm using MEGA7 (77) with overall branch stability estimated with bootstrap analysis (1,000 replications).

ABRicate (v. 0.8.10) (78) was used for the mass screening of virulence using VFDB with sequence identity and alignment coverage of >80% (25). Further identification of O and H groups (SeroTypeFinder, v2.0) (17), AMR genes (MEGARes, v.2.0) (79), and a more targeted curated subset of E. coli virulence genes, including those from extraintestinal pathogenic E. coli (VirulenceFinder, v.2.0.3) (26, 27), was undertaken by batch uploading assembled genome fasta files to the Center for Genomic Epidemiology web resource. Phylotyping of assembled genomes was carried out using the ClermonTyper web interface (80).

Statistical analysis was performed using R version 3.3.1 (81), and figures were produced using the package ggplot2 (82). To test the effects of site (site 1 to site 5) on RT-PCR targeting the presence or absence of *stx*_1_, *stx*_2_, and *eae* genes from each enrichment boiled lysate preparation, lme4 (83) was used to fit a binomial generalized mixed effect linear model on the RT-PCR presence or absence allelic data from each enrichment boiled lysate preparation. Individual “visit” was included in the model as a random effect. Linear mixed effects models were also used to examine the relationship between E. coli MPN/100 mL concentrations and the presence/absence of *stx*_1_, *stx*_2_, and *eae* genes using RT-PCR and to compare the loading of E. coli cells per gram dry weight of sediment and soil samples. Further details on sampling sites and methodology are available in the accompanying supplemental material.

### Data availability.

WGS data (238 bacterial isolates) have been deposited to NCBI under BioProject number PRJNA576546 (SAMN12996327 to SAMN12996568).
