# Supplementary material for: Whole-Genome Sequencing and Virulome Analysis of Escherichia coli Isolated from New Zealand Environments of Contrasting Observed Land Use
Source: Appl Environ Microbiol. 2022 Apr 20;88(9):e00277-22. doi: 10.1128/aem.00277-22 (PMC9088250; doi:10.1128/aem.00277-22)
Supplement: Supplemental file 4 — Supplemental methods. Download aem.00277-22-s0004.pdf, PDF file, 0.2 MB [file aem.00277-22-s0004.pdf]

Supplemental Information

Whole genome sequencing and virulome analysis of *Escherichia coli* isolated from New Zealand environments of contrasting observed land use.

Adrian L. Cookson[1,2], Jonathan C. Marshall[2,3], Patrick J. Biggs[2,4,5], Lynn E. Rogers[1], Rose M. Collis[1,2], Megan Devane[6], Rebecca Stott[7], David A. Wilkinson[2,5], Janine Kamke[8], Gale Brightwell[1,5]

[1] Food Systems Integrity, AgResearch Limited, Hopkirk Research Institute, Massey University, Palmerston North, New Zealand

[2] mEpiLab, School of Veterinary Science, Massey University, Palmerston North, New Zealand

[3] School of Mathematics and Computational Sciences, Massey University, Palmerston North, New Zealand

[4] School of Natural Sciences, Massey University, Palmerston North, New Zealand

[5] New Zealand Food Safety Science and Research Centre, Massey University, Palmerston North, New Zealand

[6] Environmental Science and Research Limited, Christchurch, New Zealand

[7] National Institute of Water and Atmospheric Research, Hamilton, New Zealand

[8] Horizons Regional Council, Palmerston North, New Zealand

Corresponding author: Adrian Cookson, Food Systems Integrity, AgResearch Limited, Hopkirk Research Institute, Massey University, Cnr University Avenue and Library Road, Private Bag 11008, Palmerston North, 4442, New Zealand. Email: adrian.cookson@agresearch.co.nz Telephone: +64 (0)6 351 8681

Running title: *Escherichia* spp. recovery from the environment

This Supplemental Information PDF file contains:

Supplemental Experimental Procedures with references.

Experimental Procedures

Sampling

Five sites in the North Island of New Zealand were sampled in this study on five or six occasions over one year. Four sites (Site 1 to 4) were from the Manawatū River (235 km long) catchment comprising an area of 5898 km^2 with an Eastern and Western catchment, separated by the Tararua/Ruahine mountain ranges. Site 1 is a headwater stream site within Pūkaha Mount Bruce (www.pukaha.org.nz), an intensively managed conservation reserve where trapping of introduced predatory species (mustelids, possums, hedgehogs and rodents) is undertaken to enhance native biodiversity. This site is within closed cover native forest, and free of endemic and introduced waterfowl species. Site 2 is 21.5km (direct distance) downstream from the Pūkaha Mount Bruce headwater site at the Hamua Bridge (Mākākahi River site, Tararua), where sheep and beef farming operations form much of the adjacent land use.

Sites 3 and 4 are at the confluence of the Mangaterā and Mākirikiri Streams (Tararua), south of Dannevirke (population 5200). Livestock farming (sheep, beef, dairy) also dominated the catchment land use for both these streams. Both streams were sampled; sampling Site 3 on the Mākirikiri Stream was immediately downstream from a small stand of native forest and 30m upstream of the confluence with the Mangaterā Stream, whilst sampling Site 4 on the Mangaterā was about 50m upstream of the confluence with the Mākirikiri Stream.

Samples were also obtained from a constructed wetland in the Toenepi River catchment at Kiwitahi near Hamilton in the Waikato (Site 5). The wetland is rain-fed and intercepts tile drainage flow from intensively grazed dairy pasture (1). The Toenepi constructed wetland is a two-stage surface-flow wetland with an area of 260m^2 (about

1% of the contributing drainage area) and is planted predominantly with raupō bullrush (*Typha orientalis*).

At each site water, sediment (not Site 2), soil, periphyton and fecal samples were obtained. All samples were kept at 4°C and processed on the day of collection. Water samples were taken from the top of the water column and *E. coli* enumerated using Colilert-18 and Quanti-Tray/2000 (IDEXX, ME, USA). Trays were incubated at 35°C (18 to 21 hours) (2) and UV fluorescent wells were enumerated to provide an indication of *E. coli* counts. *E. coli* were recovered by filtering water samples (100ml) through 0.22μm nitrocellulose filters using positive pressure, placing the filter on CHROMagar™ ECC plates (CHROMagar Microbiology, Paris, France) and incubating at 35°C (18 to 21 hours). Blue colonies were sub-cultured, incubated at 35°C (18 to 21 hours) and growth resuspended in EC broth containing glycerol (33% [w/v]) for storage at -85°C. A crude DNA extraction from each isolate was carried out by resuspending bacterial material in 400μl sterile MilliQ water, heating at 100°C for 10min with subsequent storage at -20°C. A further 100ml water sample was filtered as before with the filter enriched in 10ml EC broth at 35°C (18 to 21 hours). A crude boiled lysate of the enrichment preparation for subsequent DNA analysis was carried out by centrifuging (13,500 x g, 5min) broth culture using a bench-top microcentrifuge. The cell pellet was the washed with phosphate buffered saline (0.01M PBS, pH 7.3), resuspended in sterile MilliQ water, boiled in a heating block at 100°C and stored at -20°C. Matched water samples collected on the same day from the Site 2 and Site 4 by regional authorities during routine freshwater quality assessments, were also included in the study and were processed on the same day using the same methodology described above.

Freshwater sediment samples were obtained using a stainless-steel shovel and sieved through a mesh size of approximately 3mm to retain coarse particles and excess liquid removed to leave solid material only. Adherent bacteria were removed from the sieved sediment (10g) using 90ml peptone (0.1%) saline (0.85%) extractant (3) in a 250ml Schott bottle and shaken gently by hand for 1min. The material was left for 10min to settle before supernatant material was removed for subsequent bacterial analyses. *E. coli* were enumerated using Colilert-18 and Quanti-Tray/2000 by diluting 1ml sediment supernatant material in 99ml sterile MilliQ water and incubating at 35°C (18 to 21 hours). Bacteria were recovered using filtration by diluting 1ml supernatant in 99ml sterile MilliQ water and filtering with positive pressure through a nitrocellulose filter as described above. Filters were placed onto CHROMagar™ ECC plates and incubated at 35°C (18 to 21 hours). Finally, 1g sediment material was enriched in 9ml EC broth by incubating at 35°C (18 to 21 hours). Broth culture (10μl) was inoculated onto CHROMagar™ ECC plates, streaked for individual colonies and incubating at 35°C (18 to 21 hours). Blue colonies from CHROMagar™ ECC plates were sub-cultured and incubated at 35°C (18 to 21 hours).

Soil sample sites were collected at a distance of 5 to 10m from freshwater sample sites. A composite soil sample free of vegetation from each site (~70g) was obtained using a sterile 150mm stainless steel corer and processed for the enumeration and recovery of *E. coli* as described previously for sediment samples except that a separate extractant was used; a 1% (w/v) gelatin preparation in water was adjusted to pH 10.3 and hydrolysed by autoclaving (121°C 15lb/in^2) for 10min (4). A 1:10 dilution of the gelatin preparation in 0.1M $(NH_4)_2HPO_4$ was then used as the extractant.

The dry weight of sediment and soil samples was determined by weighing 20g material into pre-weighed foil trays. Sediment and soil samples were baked at 105°C for 24 hours with the samples weighed again to calculate moisture content and to establish the number of *E. coli* present per g dry weight of soil/sediment using Quanti-Tray/2000 data.

Periphyton samples were obtained from all stream sites by carefully removing a fully submerged rock from the waterway and wiping an area of approximately $100cm^2$ using a sterile sponge swab (EZ-Reach Sponge Sampler, World Bioproducts, Washington, USA). The sterile sponge swab was stomached for 1min with 25ml EC broth (Oxoid, Hampshire, UK) and incubated at 35°C (18 to 21 hours). Broth culture (10μl) was inoculated onto CHROMagar™ ECC plates, streaked for individual colonies and incubated at 35°C (18 to 21 hours).

For all sample types, the growth from four separate sub-cultured colonies from each sample preparation were stored at -80°C by resuspending individual colonies in EC broth containing glycerol (33% [w/v]). Crude boiled DNA isolate and enrichment extracts from all samples were performed and stored at -20°C.

Opportunistic fecal samples were was obtained using a sterile Amies swab (Copan Diagnostics Inc., Brescia, Italy) or sterile specimen container with scoop cap. Fecal material was diluted 1:100 in EC broth and incubated at 35°C (18 to 21 hours). Bovine and ovine fecal enrichments were inoculated with a composite sample obtained by combining fecal material recovered from 3 well-separated fecal deposits, where available. Broth culture (10μl) was inoculated onto CHROMagar™ ECC plates, streaked for individual colonies and incubated at 35°C (18 to 21 hours). Crude DNA

isolate and enrichment extracts from fecal material were performed and stored as described above.

The Pūkaha Mount Bruce reserve access point to reach Site 1 required crossing through a beef cattle grazing area where opportunistic collection of cattle, rabbit, and cat feces occurred approximately 1.5km from the native forest sampling site. At Site 2, fecal samples were obtained from sheep and horses that were able to graze to the river edge. Grazing cattle were also present at Sites 3 and 4; bovine fecal samples were obtained from a paddock directly north and adjacent to the confluence, and from a grazed paddock on the river terrace to the south-west of the confluence. Fecal samples from wildfowl (introduced Mallard, *Anas platyrhynchos*), Pūkeko (*Porphyrio porphyrio melanotus*), introduced Common Brush-Tailed Possum (*Trichosurus vulpecula*), and Norway/Ship Rat (*Rattus norvegicus*/*rattus*) were also collected at Sites 3 and 4. At the constructed wetland site (Site 5), additional samples were obtained for a further study not reported here, to examine the survival and persistence of *E. coli* along the length of the constructed wetland. Water and sediment samples were collected immediately downstream of the out-flow weir. Soil and bovine fecal samples were obtained from within 10m of the out-flow water sample site. Water and sediment samples were also obtained from immediately downstream of the central construction bund that separates the two wetland cells. Finally, water and sediment samples were collected immediately downstream from the wetland in-flow weir, and bovine faeces and soil samples were obtained approximately 10m above the weir. Submerged plant material (harakeke flax [*Phormium tenax*] and raupō bullrush [*Typha orientalis*]) were sampled at the in-flow and bund sites of the constructed wetland as no submerged rocks were present. A single avian (passerine species) fecal sample was collected at the wetland site (Site 5).

Molecular analyses and whole genome sequencing of *Escherichia* isolates

For Sanger sequencing, a 284bp partial *gnd* sequence was amplified by PCR (2gndF and 2gndR primers) and sequenced (ABI3730 DNA Analyzer, Massey Genome Service, Massey University, Palmerston North, New Zealand) as described previously (5). The crude DNA extraction from each isolate was used as PCR template and PCR products were purified using the QIAquick PCR (Qiagen, Hilden, Germany) purification kit. Separate sequencing PCRs were carried out using 2gndF and 2gndR (3.2μM) primers respectively and BigDye^{TM} Terminator (Version 3.1) polymerase (ABI, Waltham, MA, USA). Sanger sequencing separation was performed at the Massey Genome Service (Massey University, Palmerston North, New Zealand) using an ABI3730 DNA Analyzer. Respective 2gndF and 2gndR sequencing traces were processed to remove primer sequences using Geneious (v11.1.5, Biomatters Development Team) (6). The *gnd* sequence type (gST) from each *E. coli* isolate was identified using a custom-made gndDb database (7) of 615 distinct 284bp *gnd* sequences obtained from publicly-available genome sequence data.

Real-time (RT) PCR was used to detect *uid*A (β-glucuronidase), stx_1, stx_2 and *eae* using PerfeCTa® MultiPlex qPCR ToughMix® (Quanta Biosciences, Gaithersburg, MD, USA) and primers and probes specific for the *E. coli* allele (8). Boiled lysates of individual colonies or enrichment cultures was used as DNA template for RT-PCR. Positive samples were those with a C_q of <35.

Escherichia ruysiae (cryptic *Escherichia* Clades III and IV) and *Escherichia marmotae* (cryptic *Escherichia* Clade V) were identified using the PCR primers and conditions described previously (9).

For whole genome sequencing, *E. coli* were resuscitated to single colonies on Columbia sheep blood agar and a single colony sub-cultured onto fresh Columbia sheep blood agar. DNA was extracted using the QIAamp DNA mini kit (Qiagen) and the Nextera XT DNA library preparation kit (Illumina, San Diego, CA, USA) used to generate individual barcoded library preparations of DNA fragments of approximately 250-1500 bp. Individual libraries underwent a quality control check (LabChip® GX Touch HT, Perkin Elmer at the Massey Genome Service, Massey University, New Zealand) before libraries from separate *E. coli* isolates were combined (pooled) in equimolar ratios to ensure even sequencing coverage across all samples. WGS was undertaken by Novogene Limited (Beijing, China) using the Illumina HiSeq paired end v4 platform (2 x 125 bp). The nullarbor2 pipeline (10) was used to process WGS read data and contains modules to clean sequencing reads, and provide a suite of data outputs: QC analysis, species identification, *de novo* assembly (SKESA v.2.2.1) (11), and genome annotation (Prokka v.1.13.3) (12) for each input genome sequence and core genome SNP analysis (13), and core SNP phylogeny (13), per genome sequence set. Initially a SNP alignment from whole genome sequencing data was generated using Snippy (v.4.2.1) (13) and *E. coli* IAI39 (accession CU928164) as the reference genome. Subsequently the SNP alignment was imported into SplitsTree (v.4.14.8) (14) and a Maximum Likelihood phylogenetic tree (15) generated from alignment data.

Phylogenetic typing of assembled genomes was carried out using the ClermonTyper web-interface (16).

WGS data (238 bacterial isolates) has been deposited to NCBI under BioProject number PRJNA576546 (SAMN12996327-SAMN12996568).

All statistical analysis were performed using R version 3.3.1 (17) and figures were produced using the R package ggplot2 (18).

References.

1. Tanner CC, Nguyen ML, Sukias JPS. Using constructed wetlands to treat subsurface drainage from intensively grazed dairy pastures in New Zealand. Water Science and Technology. 2003;48(5):207-13.
2. Byappanahalli MN, Whitman RL, Shively DA, Sadowsky MJ, Ishii S. Population structure, persistence, and seasonality of autochthonous *Escherichia coli* in temperate, coastal forest soil from a Great Lakes watershed. Environmental Microbiology. 2006;8(3):504-13.
3. Piorkowski G, Jamieson R, Bezanson G, Truelstrup Hansen L, Yost C. Reach specificity in sediment *E. coli* population turnover and interaction with waterborne populations. Science of the Total Environment. 2014;496:402-13.
4. Kingsley MT, Bohlool BB. Release of *Rhizobium* spp. from tropical soils and recovery for immunofluorescence enumeration. Applied and Environmental Microbiology. 1981;42:241-8.
5. Cookson AL, Biggs P, Marshall JC, Reynolds A, Collis RM, French NP, et al. Culture independent analysis using *gnd* as a target gene to assess *Escherichia coli* diversity and community structure. Scientific Reports. 2017;7:841.
6. Kearse M, Moir R, Wilson A, Stones-Havas S, Cheung M, Sturrock S, et al. Geneious Basic: an integrated and extendable desktop software platform for the organization and analysis of sequence data. Bioinformatics. 2012;28:1647-9.
7. Cookson AL, Lacher DW, Scheutz F, Wilkinson DA, Biggs PJ, Marshall J, et al. gndDb, a database of partial gnd sequences to assist with analysis of *Escherichia coli* communities using high-throughput sequencing. Microbiology Resource Announcements. 2019;8:e00476-19.
8. Anklam KS, Kanankege KST, Gonzales TK, Kaspar CW, Döpfer D. Rapid and reliable detection of Shiga Toxin–Producing *Escherichia coli* by Real-Time multiplex PCR. Journal of Food Protection. 2012;75:643-50.
9. Clermont O, Gordon D, Brisse S, Walk S, Denamur E. Characterization of the cryptic *Escherichia* lineages: rapid identification and prevalence. Environmental Microbiology. 2011;13(9):2468-77.
10. Seemann T, Goncalves da Silva A, Bulach DM, Schultz MB, Kwong JC, Howden BP. Nullarbor - pipeline to generate complete public health microbiology reports from sequenced isolates 2019 [Available from: https://github.com/tseemann/nullarbor.
11. Souvorov A, Agarwala R, Lipman DJ. SKESA: strategic k-mer extension for scrupulous assemblies. Genome Biology. 2018;19:153.
12. Seemann T. Prokka: rapid prokaryotic genome annotation. Bioinformatics. 2014;30(14):2068-9.
13. Seemann T. Snippy version 4.2.1 2018 [Available from: https://github.com/tseemann/snippy
14. Huson DH, Bryant D. Application of phylogenetic networks in evolutionary studies. Journal of Molecular Biology. 2006;23(2):254-67.
15. Kozlov AM, Darriba D, Flouri T, Morel B, Stamatakis A. RAxML-NG: a fast, scalable and user-friendly tool for maximum likelihood phylogenetic inference. Bioinformatics. 2019;35(21):4453-5.
16. Beghain J, Bridier-Nahmias A, Le Nagard H, Denamur E, Clermont O. ClermonTyping: an easy-to-use and accurate in silico method for *Escherichia* genus strain phylotyping. Microbial Genetics. 2018;4:DOI 10.1099/mgen.0.000192.
17. R Core Team. R: A language and environment for statistical computing Vienna, Austria2018 [Available from: https://www.R-project.org.
18. Wickham H. ggplot2: Elegant Graphics for Data Analysis. 2nd Edition ed: Springer Publishing Company, Incorporated; 2009.
